# Thrombosed Aneurysm of Superficial Epigastric Vein Simulating Inguinal Hernia: Report of Two Cases

**DOI:** 10.1155/2021/2418863

**Published:** 2021-10-04

**Authors:** Eleni Skandalou, Panagiotis Papadopoulos, Marianthi Kavelidou, Stavros Kalfadis, Theodoros Tzigkalidis, Ioannis Skandalos

**Affiliations:** ^1^Internal Medicine Department, General Hospital “G. Papanikolaou”, Thessaloniki, Greece; ^2^Surgical Department, General Hospital “Agios Pavlos”, Thessaloniki, Greece; ^3^B΄Surgical Department General Hospital “G. Papanikolaou”, Thessaloniki, Greece; ^4^Pathology Department, General Hospital “Agios Pavlos”, Thessaloniki, Greece

## Abstract

**Aim:**

Presentation of two cases of superficial epigastric vein aneurysm simulating inguinal hernia. To our knowledge, only one other case is reported in the literature. *Case presentation*. The first case was a 34-year-old female with left inguinal pain and swelling which was clinically diagnosed as inguinal hernia. The second case was a 28-year-old female with inguinal pain and swelling, depicted with triplex ultrasonography and computed tomography, and was suspected to have inguinal hernia or enlarged inguinal lymph node. During the surgical exploration, both patients were found to have thrombosed aneurysm of the superficial epigastric vein. During the surgical exploration, both patients were found to have thrombosed aneurysm of the superficial epigastric vein. The superficial epigastric vein was ligated, and the venous aneurysms (6 × 4 × 3 and 2 × 3 × 2.5 *cm*, respectively) were excised. Histological examination of the thrombosed aneurysm showed complete replacement of the vascular wall by fibrous tissue, thrombosis, and an inflammatory reaction. There were no postoperative complications, and both patients were discharged on the second postoperative day. The 3-month and 1-year follow-up examination, respectively, was uneventful.

**Conclusion:**

Although venous aneurysms in the inguinal area are rare, they should be included in the differential diagnosis of a groin swelling.

## 1. Introduction

Venous aneurysms are usually uncommon. Venous aneurysms were first identified in autopsy studies by Osler in 1915 (1). Venous aneurysms can be caused by trauma, inflammation, connective tissue abnormalities, and degenerative changes (2). Superficial venous aneurysms in the inguinal region may be misdiagnosed soft tissue masses or inguinal or femoral hernias (3, 4). Herein, we describe two cases of a thrombosed aneurysm of the superficial epigastric vein simulating an inguinal hernia. To our knowledge, only one other case of inferior epigastric vein aneurysm is reported in the literature, and it is not clear if this is an aneurysm of the superficial or the deep inferior epigastric vein (5).

## 2. Case Presentation

First case: a 34-year-old female patient was admitted for an extensive painful swelling along her left inguinal area during the past 6 months. She was a nonsmoker, there was no trauma, use of oral contraceptive pills, infection, inflammatory disease, and recent travel in her medical history while review of systems was unremarkable. The patient had a medical history of left saphenectomy 8 years ago. Physical examination revealed a nonpulsating, immobile, and painful swelling along her left groin. The results of electrocardiography, routine biochemistry, and blood analysis were normal. The patient was clinically diagnosed with a left inguinal hernia, while an ultrasonic scan, unfortunately, had not been previously performed.

**Figure 1:**
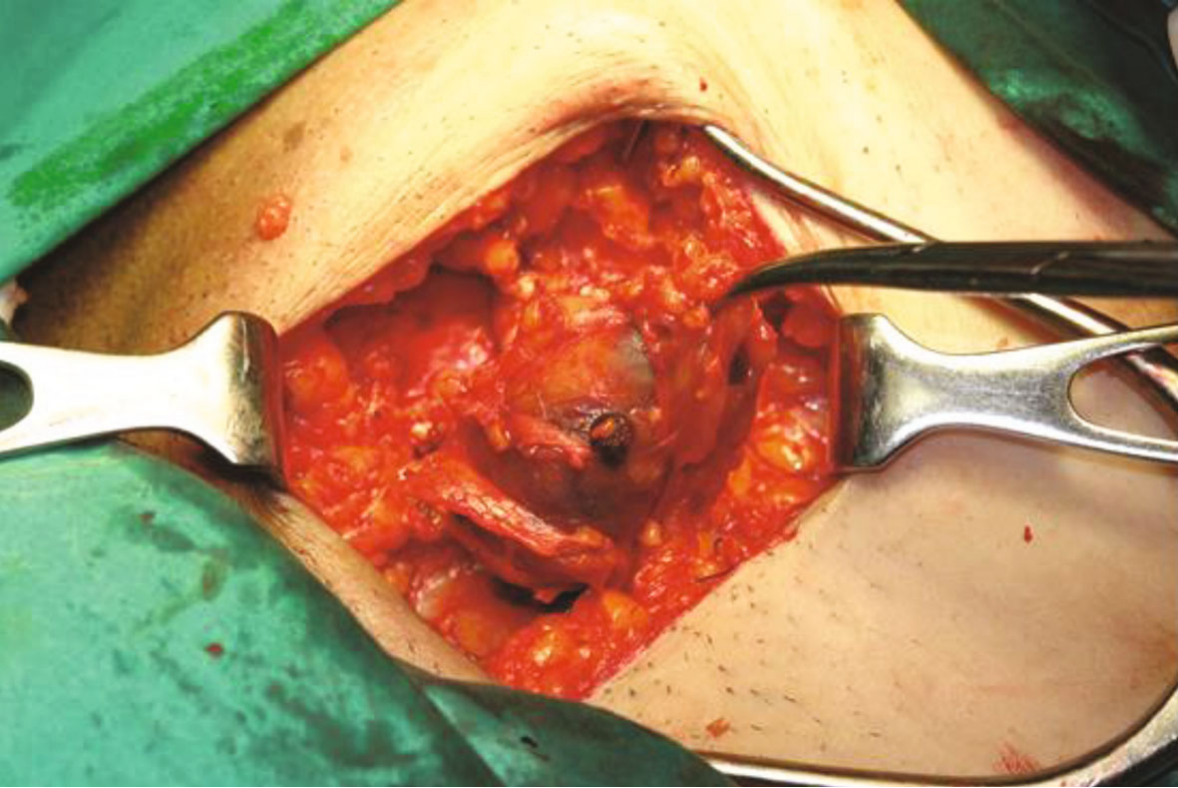
Surgical field: thrombosed aneurysm of the left superficial epigastric vein.

**Figure 2:**
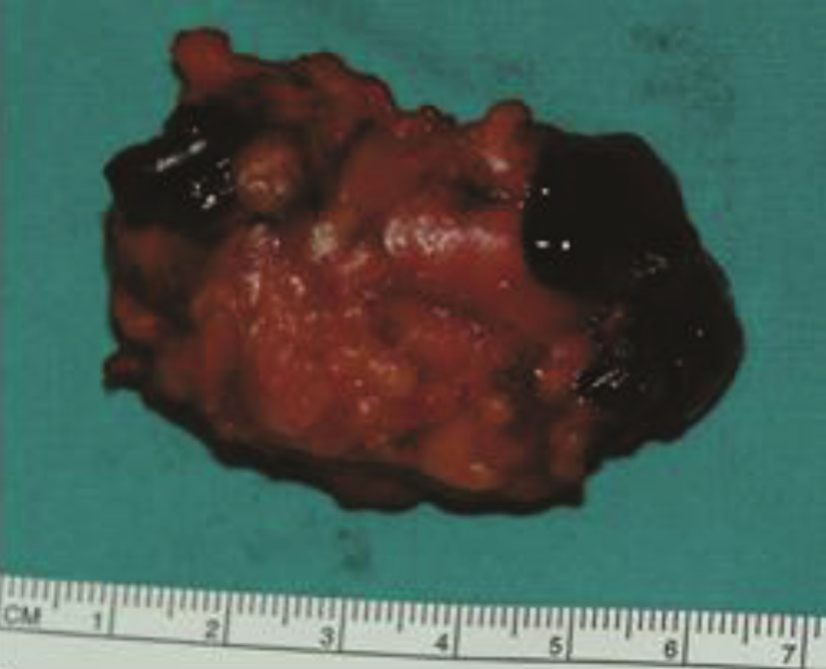
Surgical specimen: resected thrombosed superficial epigastric venous aneurysm (6 × 4 × 3 cm).

**Figure 3:**
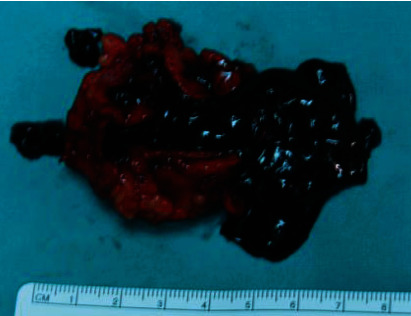
Surgical specimen: esected thrombosed superficial epigastric venous aneurysm (6 × 4 × 3 cm).

The patient was operated under spinal anesthesia, and a left oblique inguinal incision was performed. A large blue mass, due to a thrombosed fusiform aneurysm of the superficial epigastric vein, was found (Figure 1). The superficial epigastric vein was ligated flat to saphenous stump, and the venous aneurysm (6 × 4 × 3 *cm*) was excised (Figures 2 and 3).

**Figure 4:**
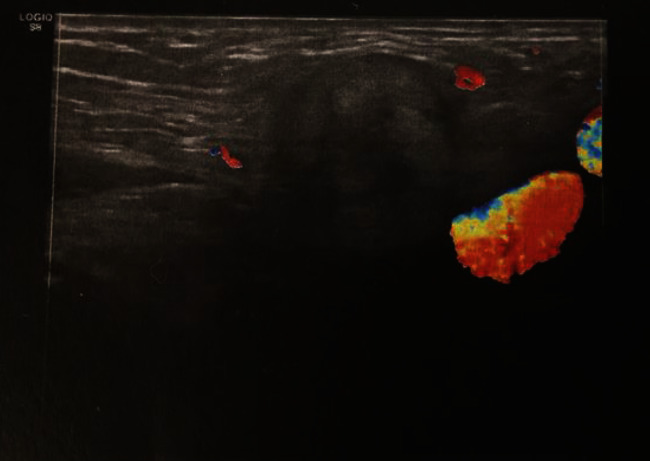
Tripplex ultrasonography: ultrasound findings suggesting left inguinal hernia (arrow).

**Figure 5:**
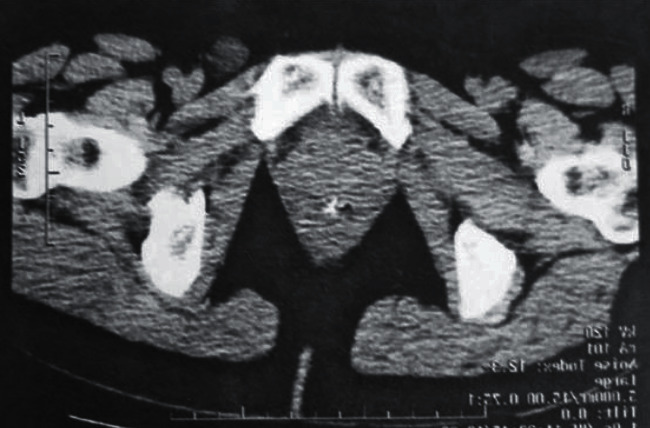
Computed tomography: in the inguinal region, a clearly demarcated formation diameter of 2 cm, with solid and cystic elements, is showed (swollen lymph node?) (arrow).

The second case: a 28-year-old female patient was admitted for mild painful swelling along the left inguinal area over the past 2 months. There were no remarkable findings in the patient's history of the predisposing factors, and the review of the systems was unremarkable. Physical examination revealed an inconsolable, immobile, and painful swelling along the left groin. The imaging examination with triplex ultrasonography (left inguinal hernia) (Figure 4) and computed tomography (inguinal lymph node enlargement) (Figure 5) was not diagnostic. The results of electrocardiography, biochemistry routine, and blood analysis were normal.

**Figure 6:**
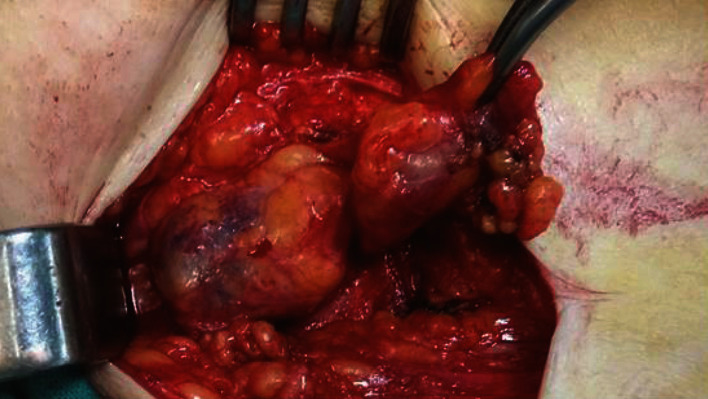
Surgical field: thrombosed superficial epigastric venous aneurysm (white arrow) and lymph node (black arrow).

**Figure 7:**
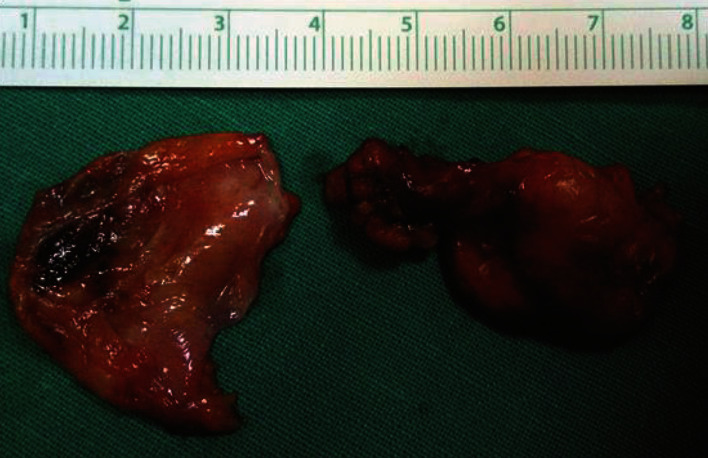
Surgical specimen: thrombosed superficial epigastric venous aneurysm (2 × 3 × 2.5 cm) (white arrow) and lymph node (black arrow).

The differential diagnosis preoperatively was left inguinal hernia and inguinal lymph node enlargement. The patient was operated under spinal anesthesia, and a left oblique inguinal incision was performed. A large blue mass, due to a thrombosed saccular aneurysm of the superficial epigastric vein, was found (Figure 6). The superficial epigastric vein was ligated flat to saphenous stump, and the venous aneurysm (2 × 3 × 2.5 *cm*) was excised (Figure 7).

## 3. Results

**Figure 8:**
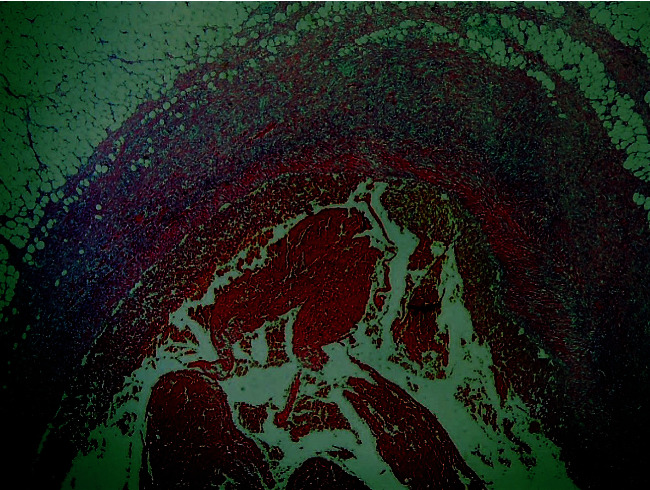
Complete replacement of three normal wall layers by fibrous tissue (white arrow), with thrombosis (blue arrow) and inflammatory reaction (black arrow) (H&E ×20).

Histological examination of the thrombosed aneurysm showed complete replacement of the vascular wall by fibrous tissue, thrombosis, and an inflammatory reaction (Figure 8). There were nopostoperative complications, and both patients were discharged on the second postoperative day. The 3-month and 1-year follow-up examination, respectively, was uneventful.

## 4. Discussion

Venous aneurysms are rare vascular malformations that occur throughout the body and vessels at various sites can be affected as in the internal jugular vein, superior vena cava, inferior vena cava, superior mesenteric vein, and also veins of the extremities (6, 7). Primary venous aneurysms are morphologically divided into two subgroups, saccular and fusiform (8). In literature, there are cases of venous aneurysms in all ages with equal distribution between both sexes (9). Primary venous aneurysms are usually congenital or develop from defective venous wall tissue (10). Secondary or acquired venous aneurysms are usually found in adults and are associated with trauma, inflammation, stretch injury, or altered venous hemodynamics. The previously reported case of superficial epigastric vein thrombosed aneurysm (5) and our two cases occurred in women of young age.

Histologically, there is replacement of three normal vascular wall layers by fibrous tissue with thrombosis and inflammatory infiltration (11). In both of our cases, the histological findings, due to aneurysm thrombosis, were complete replacement of the vascular wall by fibrous tissue, thrombosis, and an inflammatory reaction.

The causing symptoms are due to disfigurement, rupture, fistulation, or compression. Deep venous aneurysms present with pain or pulmonary embolism, while superficial venous aneurysms are usually asymptomatic; so, venous aneurysms located in the inguinal region,could be misdiagnosed as soft tissue lesions (6, 12) or as inguinal hernias (3, 4). Patients often complain of pain and a gradually increasing swelling (12). Because of the confusing symptoms and the unknown clinical entity, our patients were clinically misdiagnosed as having an inguinal hernia instead of the correct diagnosis.

Venous aneurysms can generally be diagnosed mainly by color Doppler imaging (12), as well as by computed tomography (CT) or magnetic resonance imaging (MRI). Our second patient was misdiagnosed by imaging examination with triplex ultrasonography (left inguinal hernia) and computed tomography (inguinal lymph node enlargement), due to the rarity and not considering of the diagnosis of superficial epigastric vein aneurysm.

The anatomic position of the venous aneurysm defines the best surgical approach. The indication for surgical treatment of superficial, neck, and face venous aneurysms is usually cosmetic. Asymptomatic nonenlarging venous aneurysms can be safely followed up, due to their high surgical morbidity and mortality. Abdominal venous aneurysms are at high risk of becoming painful or bleeding, and thus surgery should be considered for low risk patients (7, 13). As deep venous aneurysms of the extremities are at high risk of thromboembolic complications despite anticoagulant therapy, surgery may be the optimal management in terms of tangential aneurysmectomy and lateral venorrhaphy (9). Endovascular techniques continue to lack a defined role in their management (6). A superficial epigastric venous aneurysm is a rare entity. It should be suspected in cases of an inguinal mass, and it can be safely resected, because it is a superficial vein.

## 5. Conclusion

We report two rare cases of a thrombosed venous aneurysm of the superficial epigastric vein, simulating an inguinal hernia. Although venous aneurysms in this region are rare, they should be included in the differential diagnosis of a groin swelling.
